# Experiences of family building counseling and perceptions of reproductive technology among adults with sickle cell disease

**DOI:** 10.1371/journal.pone.0355020

**Published:** 2026-08-24

**Authors:** Rachel D. Gordon, Kayla Dyson, Nives Quaye, Leena Nahata, Susan E. Creary, Robert M. Cronin

**Affiliations:** 1 Department of Internal Medicine, The Ohio State University College of Medicine, Columbus, Ohio, United States of America; 2 The Ohio State University College of Medicine, Columbus, Ohio, United States of America; 3 Division of Endocrinology, Nationwide Children’s Hospital, Columbus, Ohio, United States of America; 4 Division of Hematology/Oncology/BMT, Nationwide Children’s Hospital, Columbus, Ohio, United States of America; King Fahd Military Medical Complex, SAUDI ARABIA

## Abstract

**Introduction:**

Sickle cell disease (SCD) is an autosomal recessive blood disorder that impacts about 100,000 Americans. With increasing life expectancy and more people with SCD reaching reproductive age, there is growing evidence that SCD and some of its therapies decrease fertility. While partner testing and assisted reproductive technology (ART) can enable people with SCD to choose if and how to have biological children, their use remains limited. In this study, we aimed to explore how people with SCD understand the potential fertility implications of SCD and their perceptions of partner testing and ART.

**Methods:**

We recruited adults with SCD ages 18–35 years old who self-identified as being interested in having a biological child in the future and conducted semi-structured individual interviews to discuss previous experiences and perceptions regarding family building planning. Interviews were transcribed and thematically analyzed using inductive coding and the social ecological model.

**Results:**

We completed 11 interviews with 9 women and 2 men (mean age 30.6 years old, 100% Black or African American). We identified five themes that reached saturation: 1) Providers gave limited support for family building planning; 2) Female participants had a lack of understanding what pregnancy would be like with SCD; 3) Participants felt their partners did not equally share the burden of family building planning; 4) Potential financial burden prevented participants from seriously considering ART; and 5) Participants wanted earlier and frequent discussions with their providers about family building options. Participants identified factors that impacted their family building plans, which fit across the social ecological model.

**Discussion:**

Adults with SCD desire further education on the impact of SCD and its treatments on fertility as well as support in their reproductive planning through early, frequent conversations about family building options. Additionally, financial barriers to ART such as insurance coverage must be addressed for adults with SCD.

## Introduction

Sickle cell disease (SCD) is an autosomal recessive hemoglobinopathy affecting 100,000 individuals in the United States that leads to chronic pain and end-organ damage, including retinopathy, nephropathy, splenic infarction, and pulmonary hypertension [1]. With improving treatment options, the average life expectancy at birth has increased from less than 20 years in the 1970s [2] to 54 years in 2019 [3]. Therefore, more people with SCD are reaching reproductive age [4] and will need to consider the impact SCD has on their reproductive health. For instance, people with SCD have a 50% chance of having a child with SCD if their partner has sickle cell trait (SCT), which is present in an estimated 8% of African Americans [1]. Despite universal newborn screening [5] and recommendations for counseling, [6] knowledge of individual SCT status often remains limited [7]. Thus, testing partners of people with SCD for abnormal hemoglobin traits that could lead to SCD is critical to understand the couple’s risk of having a child with SCD and allow them to make informed decisions in regards to family building and the planning of if, when, and how to have children [8].

Beyond genetic transmission, SCD has additional reproductive implications [9]. There is growing concern that SCD and some of its treatments may lead to decreased fertility, including new curative therapies [10]. Hypoxic injury from SCD may lead to premature gonadal failure; women with SCD have higher rates of primary ovarian insufficiency [11] and men with SCD have been shown to have abnormal semen parameters [12]. Hydroxyurea, a common treatment for SCD, has been shown to decrease sperm counts [13], but it is unclear if these effects are transient [14]. Conditioning regimens for hematopoietic stem cell transplantation and gene therapy can also significantly impact fertility [15,16]. Given that adolescents and young adults with SCD indicate that biological parenthood is important to them [17], increased consideration of fertility preservation and augmentation is necessary. However, only 4% of youth with SCD have been counseled on potential fertility impact of SCD [18] and in one survey, less than 50% of adults with SCD were able to identify infertility risk factors [19]. Assisted reproductive technologies (ART) have the potential to address both genetic transmittance and infertility impacts of SCD [20]. In vitro fertilization (IVF) can successfully be used in people with SCD [21], and use of IVF with preimplantation genetic testing (PGT) can ensure the resultant child does not have SCD if their partner has an abnormal hemoglobin trait [22], if desired by the family.

While it is known that most parents of children with SCD would consider using PGT for future pregnancies [23], the perceptions and opinions of adults with SCD regarding reproductive technology such as partner testing and IVF/PGT are relatively unknown. Previous work has focused mostly on knowledge of fertility and ART [18,19], but not patient-identified needs in family building counseling or intentions regarding ART if it were available. Experiences of family building decision making are multi-faceted and shaped by interpersonal and community factors across the social ecological model [24] and require in-depth understanding. In this study, we aimed to understand previous experiences with fertility counseling, perspectives on partner testing, and attitudes towards ART amongst adults with SCD.

## Methods

The study was approved by the Ohio State University (OSU) Institutional Review Board and all participants provided written informed consent for interview video recording (IRB ID 2023B0122). The consolidated criteria for reporting qualitative research (COREQ) [25] guided the reporting of this qualitative study (S1 File).

### Setting and recruitment

The study was conducted at an academic, tertiary care Midwestern medical center with a sickle cell center. A National Alliance of Sickle Cell Centers affiliate [26], the included center treats an estimated 400 adults with SCD and has obstetric providers, including specialists in Reproductive Endocrinology and Infertility (REI), within the medical center. Participants were recruited from clinic in a convenience sampling approach from February 2024 to September 2024. Patients were eligible if they had a diagnosis of SCD (any genotype) and were between 18–35 years old. Potentially eligible patients were identified from clinic schedules; these patients were then asked, “Are you considering or planning on having biological children in the future?” If the patient said yes or was unsure, they were considered eligible for participation. Exclusion criteria included inability to speak conversationally in English and unwilling to discuss fertility planning. Participants who completed the interview were compensated for their time with a $50 gift card.

### Data collection

Demographics and medical history were collected in person and securely managed using REDCap electronic data capture via the OSU [27,28]. Participants completed one-on-one video interviews that were recorded with one of two study members- a female physician trained in Internal Medicine-Pediatrics (RDG) or a female medical student (KD). They both had previous experience with qualitative research (RDG completed didactic coursework in qualitative analysis and had completed qualitative studies regarding postpartum primary care; KD conducted qualitative analysis for her Master of Public Health [MPH] thesis and was under supervision of RDG) and did not have any therapeutic relationship with the participants. Interviewers reported no existing biases and were interested in increasing knowledge regarding and access to ART for those who are interested. Participants were informed that the interviewers were researchers learning about the experiences and opinions of adults with SCD about partner testing for sickle cell trait and reproductive planning. The interviewers used a semi-structured interview guide that included questions regarding their understanding of the impact of SCD on fertility, previous counseling on reproductive options, experience with partner testing, understanding of IVF, and perceptions of PGT which had been pilot tested with two individuals (S2 File). Since it was anticipated that not all participants would have prior knowledge of IVF and PGT, brief written descriptions of these interventions were provided to all participants during the interview. Participants were recruited until thematic saturation was reached. Interviews were conducted via video at the participant’s home; non-participants were occasionally present at the discretion of the participant (e.g., children, sibling) but were not directly asked questions or engaged in the interview. Video interviews were recorded, which participants explicitly consented to. Repeat interviews, field notes, transcript returning, and participant checking were not used.

### Data analysis

**Fig 1:**
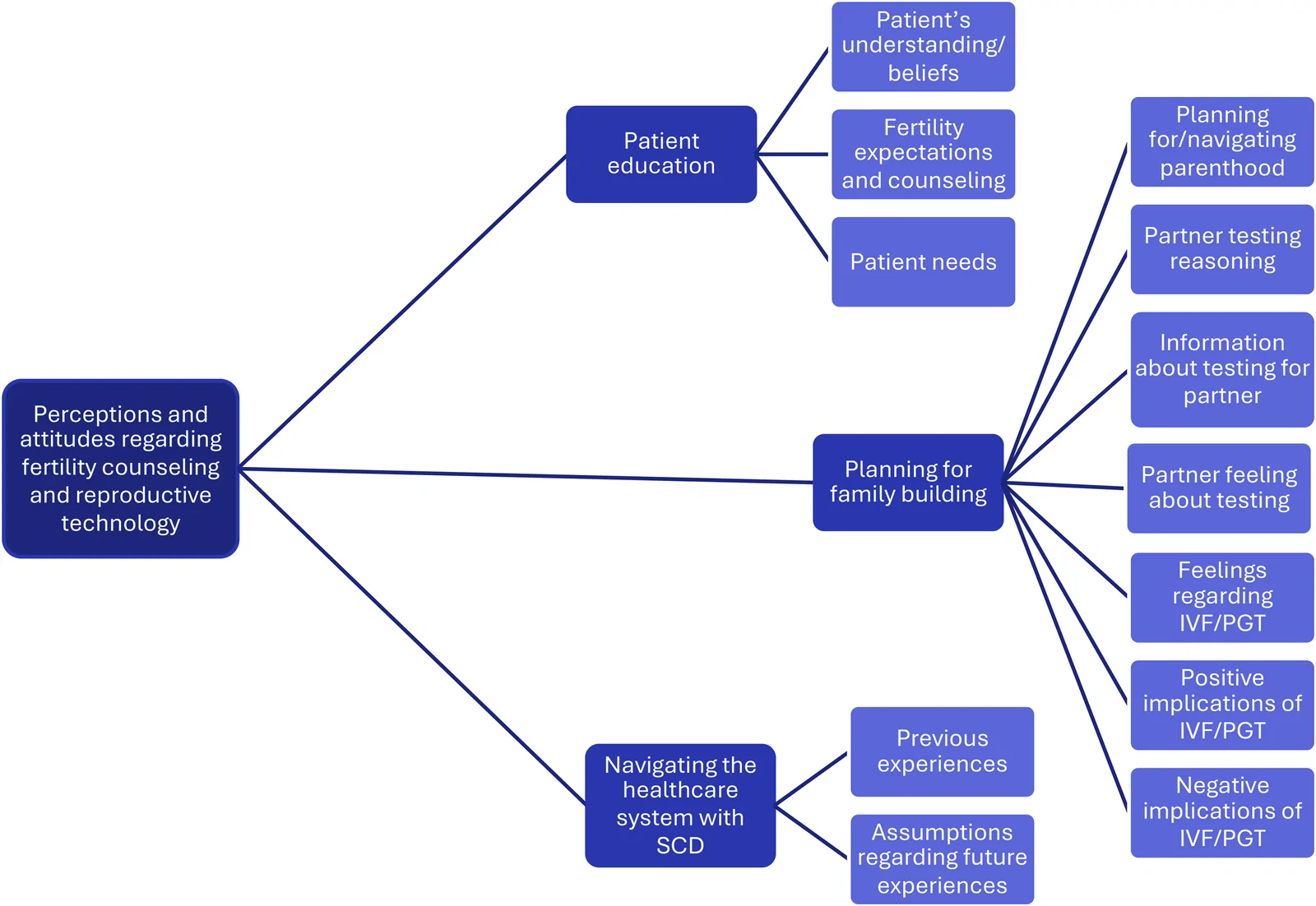
Code tree.

Analysis was led by grounded theory and narrative inquiry [29]. Interview audio recordings were transcribed and analyzed using NVivo software [30]. Two study members (RG, KD) with previous coding experience formed the coding team. Both coders first conducted line-by-line coding on two transcripts independently, then developed an initial codebook based on the codes as well as the primary research questions. They applied the shared codebook to one transcript, then compared and discussed any necessary changes to the codebook until consensus was reached. Both coders independently coded the remaining transcripts with the codebook. Codes were later grouped by applied thematic analysis (Fig 1). Data saturation was considered reached when there were no new themes identified regarding participants’ perceptions of family building planning conversations with providers; data saturation was reached by the final interview.

Transcripts were reviewed for participant-identified factors impacting their reproductive intentions which were mapped to the social ecological model. Initially described over 25 years ago to direct development of multi-level health promotion interventions [31], the social ecological model details determinants of health behaviors across five domains: individual, interpersonal, organizational, community, and public policy. Previous literature has used the social ecological model to understand influencing factors on reproductive intentions [24,32] to describe the features that lead to stratified reproduction, or socio-economic imbalances in ability to have and care for children [33]. Given the significant racial and socio-economic disparities in ART access [34], the social ecological model serves as an optimal framework to understand the overlapping dynamics that lead to individual decisions on family building.

## Results

A total of 22 female patients and 11 male patients were approached to participate. While 21 patients (15 female, 6 male) consented to participate, in total, 11 completed an interview. Of the ten participants who consented but did not complete an interview, five were not reachable to schedule an interview, and five scheduled an interview but did not show up for the interview; none of the participants dropped out. Participant demographics and disease characteristics are included in Table 1; there were nine women and two men, aged 26–34 years. All participants identified as straight/heterosexual, and three participants (all women) had biological children. Interviews lasted an average of 34 minutes (SD 12 minutes). We identified five themes through our interviews: 1) Providers gave limited support for family building planning; 2) Female participants had a lack of understanding what pregnancy would be like with SCD; 3) Participants felt their partners did not equally share the burden of family building planning; 4) Potential financial burden prevented participants from seriously considering ART; and 5) Participants wanted earlier and frequent discussions with their providers about family building options (Table 2).

**Table 1:** Participant demographics and previous experiences/perfections of reproductive technology.

Participant characteristics	N (%)
Age in years, mean (SD)	30.6 (2.8)
Female	9 (82%)
Black/African American	11 (100%)
Hispanic/Latinx	1 (9%)
Straight/heterosexual	11 (100%)
Living with partner/married	4 (36%)
Medicaid insurance	5 (45%)
Hemoglobin SS	5 (45%)
Prescribed hydroxyurea	4 (36%)
Currently have biological children	3 (27%)
**Experience and perceptions**	**N (%)**
Have asked partner’s status	8 (73%)
Would ask partner’s status	11 (100%)
Would change reproductive goals based on partner’s status	6 (55%)
Heard of IVF	11 (100%)
Heard of IVF used for people with SCD/discussed with provider	1 (9%)
Would consider IVF	4 (36%)
Heard of PGT	4 (36%)
Would consider PGT	9 (82%)

**Table 2:** Summary of themes and illustrative quotes.

Theme	Illustrative quotes
Providers gave limited support for family building planning	“I take this hydroxyurea thing. Now, the nurse practitioners are kind of telling me you know, just always use protection. But then, if you’re not wanting to and you actually want kids, then you have to like, stop taking it for 3 months. And so, I didn’t even know about the side effect till later. And I’m like well. maybe I just won’t take it. And then they’re like, “Well, then, there’s nothing to take. The other stuff don’t really work as good as that one.” So, I did that for a while, and that got me into some trouble. So yeah I’m now back on it. “ “When I was engaged, I was I was seeking out doctors to pretty much get their opinion to see one if they think that I could carry? What would that look like? And how it would affect me with sickle cell disease? But they were just pretty much telling me like, “oh, well you should probably kind of avoid it.” I have sickle SS. My health is pretty good. I try to maintain my body. But a lot of them was just like, “oh, why put yourself through that?” And then I got 3 doctors that was just like this pregnancy is probably not going to be good.” “She [hematologist] was one of the ones who was like, you know, “Just get ‘em tested so you don’t have to see a baby in pain.”” “Nobody could tell me what that looked like. Nobody, and I didn’t honestly know that it was an option for me to do IVF.” “The biggest thing that they kind of drill once you become sexually active with sickle cell is, do you know the chart? How can you get sickle cell? How is it transmitted and the different types? So, they educated on that. But as far as pregnancy I didn’t feel supported from them.” “I don’t ever really remember talking about fertility. Like, I remember talking about, like, the genetics part- like, if you have a child that has the trait or the sickle cell, whatever happens, but never like the actual, like, fertility part of it.”
Women lacked understanding of what pregnancy would be like with SCD	“But it wasn’t really a lot of information specifically about what they experience or what to expect in pregnancy.” “There’s a ton of information out there on pregnancy. I have a gynecologist, but if I’m asking questions, it’s more so about like what to expect with that in regards to sickle cell. So, having that input, I think, is what I would want the most, since I feel like that’d be the hardest to find elsewhere.” “I guess, like just what to expect with the pregnancy itself, or like what I would be able to do to have the smoothest pregnancy. Cause like, as far as like the genetics I get that that’s pretty feel like straightforward. But as far as like just what the pregnancy stuff is gonna be like and what I can do to make it as safe as possible if I do that is what I would want to know with regards to my sickle cell.” “I’ve never gotten the opportunity to talk to someone who cisgender that has sickle cell that also carried their child to term or had like a biological pregnancy. I feel like having that conversation with someone would put some of my fears at ease…. I feel like there’s something about like a lived experience that does a lot more to help people.”
Participants felt their partners did not equally share the burden of family building planning	“I do know a lot of the information shared with me was not necessarily shared with my partner. I always had to relay the information or the doctor’s point of view and stuff like that.” “She [partner] would do it but it has to be like the right circumstances where everything is kind of it’s frictionless, like hey, we go to this, then they poke your blood, and that’s it.” “I know that I will end up doing like a lot of the heavy lifting emotionally when I have these conversations.” “A lot of people think it’s like, “Oh, it’s just a Black disease,” or just “affects Black people” when it’s like no, it affects everyone. Like yes, Black people are the majority of the carriers, but anyone could have it, regardless of your race or ethnicity. So, even bringing up with partners of opposite race, I feel like they tend to brush it off quicker and not take it as serious.”
Potential financial burden prevented participants from seriously considering ART	“That stuff that would be like a consideration, but, like I could have barely get health insurance or afford health insurance as it is…I think if I we’re having problem having children, I’d probably go the foster route before considering IVF unless I came into a windfall of money.” “And then, as far as you know, that is one of the big issues with cost, you know, without insurance, it can cost, you know, $15,000.” “So yeah, if I had the money, or if, like health insurance covered it, then I would do it but... uh, that’s not really something I’ve thought about, because I know it’s expensive.” “That’s another big fear of mine is if they implant it in me, and I literally cannot have kids then I just wasted my money, and I have to do the whole dang thing all over again… then I am out of money and out of a baby- yeah, that’s a pretty big fear of mine.”
Participants wanted earlier and frequent discussions with their providers about family building options	“If our doctor brings it up not at a too early age but when you hit that twenties mark like, “Hey, have you thought about this” like this survey would be great when I was like 20. I wasn’t really thinking about it, but it was in my mind.” “It would be really nice to like, have broached this topic, maybe younger when I was like 18, or like 19, or even like in my early twenties.” “I’m kind of a little saddened to have this conversation with you and realize how much information I missed. But yeah, I would like to know about it in the future.” “I just want to hear I have a good shot or there’s no shot that this is going to work out. That’s what I want to know straight out of the gate.”

### Providers gave limited support for family building planning

While all participants were confident in the inheritance pattern of SCD, very few participants expressed understanding of the impact that SCD or SCD treatments may have on fertility. Many participants referenced providers drawing family trees or charts to explain the importance of partner testing, but did not recall discussions regarding fertility and family building. Participants who brought up concerns about fertility discussed their concern of infertility as a side effect of a medication or due to chronic pain, rather than as a complication of SCD directly. This lack of understanding may have reflected their previous education regarding reproductive topics.

Some participants assumed their fertility would not be affected because of their provider’s strong focus on preventing pregnancy. As one participant stated, “*I know that it [hydroxyurea] is something I couldn’t be on while pregnant…. So I don’t have any worries about it affecting me getting pregnant. I’m more worried about, like if I do get pregnant, how would I manage my crisis.*” Participants who discussed family building planning with their providers recalled their providers recommending against pregnancy and not providing alternative options if they expressed desire to conceive. This led some participants to avoid discussions regarding family building with their providers; several participants discussed having difficulty getting pregnant or suffering from repeated miscarriages but did not discuss this with their provider.

### Female participants had a lack of understanding what pregnancy would be like with SCD

Females expressed their concern with their lack of knowledge on what pregnancy may be like with SCD and the impact it would have on their health. Participants differed on whether they thought SCD would have an impact on pregnancy outcome for themselves or their child, and oftentimes conflated their SCD symptom burden with the likelihood of poor pregnancy outcomes; one participant stated it is important to prevent pain crises during pregnancy because “*if they’re not in pain, that would help lead to no birth defects or… possibly save them from having a miscarriage*.” Many had unanswered questions regarding the impact pregnancy would have on their SCD symptoms, particularly pain crises and anemia, as well as how they could optimize their health for pregnancy. Several women felt that besides their hematologist, it would also be helpful to hear directly from a person with SCD who had given birth to understand the experience of pregnancy with SCD.

### Participants felt their partners did not equally share the burden of family building planning

All participants stated they would ask any future partner their SCT status, and most participants reported that they had asked at least one of their partners previously (Table 1). Participants felt comfortable asking their providers for resources to get their partner tested. The timing of asking their partner about their SCT status was variable, but all participants described leading the conversation; some asked soon after beginning to date to decide if they should continue the relationship, some waited until they were in a serious relationship and beginning to consider having children, and some participants did not ask their partner’s SCT status until they were pregnant. This may have reflected the large variety of responses in whether their partner’s SCT status would change their family building plans. Several participants said they would proceed with having a child together but wanted to be prepared for the possibility of having a child with SCD; one participant stated, “*I don’t feel like people with sickle cell don’t deserve to be here*”. Other participants said they would pick a different partner or would not have biological children if their partner had SCT; they often cited their own or family members’ experiences with SCD and not wanting their child to have to experience that. One participant stated, *“I know the pain that I go through, and I wouldn’t wish it on my worst enemy, much less my child.”*

Of participants who had previously asked a partner about their SCT status, most felt good about these conversations. Many reflected that their partners had helped them through crises and so were aware of the potential significant impact of SCD on a person’s health. Some participants reported that their partners who had a perceived decreased risk (such as no family history or of a different race/ethnicity) were sometimes more resistant to testing. Participants also occasionally reported that their partners were sometimes resistant to getting testing done if they had already conceived; one participant recalled asking her partner refusing to get tested after she became pregnant because “*we are just going to deal with it no matter what and love him regardless*.”

### Potential financial burden prevented participants from serious considering ART

All participants were aware of IVF as a treatment for infertility; some referenced social contacts or online influencers who had undergone IVF as their primary reference. Only one participant was aware of IVF use for people with SCD, and they stated they had specifically asked their provider about this as an option. All participants were aware of cost as a barrier, which precluded them from considering IVF as a viable option. Other concerns regarding IVF included the uncertainty of success and the unique potential impacts of IVF medications on people SCD. One participant stated, *“I just don’t know if it’s something that, like, would harm my body more than it would do good.”*

In contrast, few participants had heard of PGT prior to the interview. There was variable interest in PGT; some participants expressed concerns about eugenic implications of PGT, calling it a “*slippery slope*”, while others liked that it could allow them to have a biological child without SCD.

### Participants wanted earlier and frequent discussions with their providers about family building options

**Fig 2:**
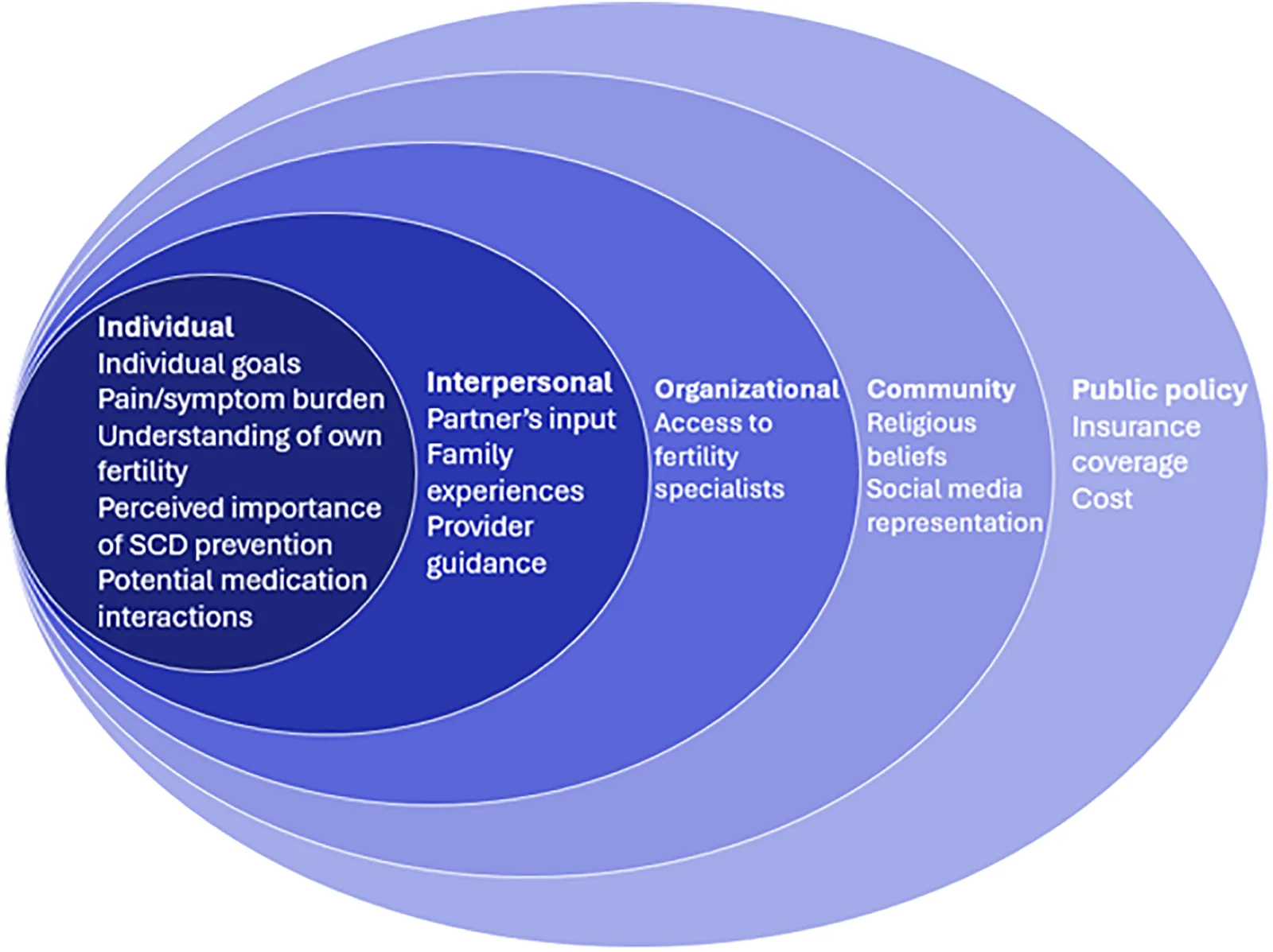
Social ecological model of identified factors affecting personal intention of reproductive technology for adults with SCD.

Participants identified wanting to have earlier and more frequent conversations about family building, with a focus on their individual goals, setting realistic expectations, and how to optimize their chances for a healthy pregnancy. Participants identified factors that would impact their family building intentions and interest in ART, which fit across the social ecological model (Fig 2), and while their provider’s recommendations did play an important role in their decision, several participants wanted to discuss family building even if their provider recommended against pregnancy. Participants largely wanted resources from their hematologist, citing longitudinal relationships and expertise in SCD as important to their discussion regarding pregnancy planning. They also reported unclear division of responsibilities within large healthcare teams, such as not understanding the role of primary care or obstetrics versus hematology and often using their hematologist as their first healthcare contact. Women who had been pregnant referenced this as one of the reasons they desired more involvement from their hematologist during pregnancy.

Participants also reported the need for resources for their partners and partners’ families to educate them regarding the potential impacts of SCD and how to get tested for SCT, both to help their partner feel more involved as well as to decrease the burden on themselves to unilaterally direct these conversations. One participant stated, “*It would be nice if he had somebody that he could talk to or felt comfortable with, like, ‘Oh, yeah... I know about the treatments,’ you know. Sometimes I can’t explain stuff to him. I don’t know how*.”

## Discussion

This study is one of the first to detail the previous experiences and perceptions of adults with SCD regarding family building intention and reproductive technology. Our study shows that not only is reproductive health and fertility counseling inadequate, in alignment with previous research [18,35], but that adults with SCD feel unsupported and not equipped to make family building decisions.

Several factors were identified that impacted participants’ family building decision making and intention to utilize reproductive technology if available (Fig 2). Providers’ guidance and individual’s understanding of their fertility, treatment effects, and disease burden were all factors in reproductive technology intention, making the guidance of healthcare providers central to allowing individuals to make the most well-informed decision based on their health status. Notably, participants differed in their value assessment of having biological children versus prevention of SCD for potential children; it is critical providers establish their patient’s priorities to tailor their counseling.

Advancements in detection and treatment have transformed the life courses of families affected by SCD; while SCD used to be considered a childhood illness due to early mortality, now 95% of people with SCD live to adulthood [1]. Subsequently, the number of people with SCD making decisions regarding family building has outpaced the current understanding of the impact of SCD on reproductive ability and pregnancy risk mitigation for women with SCD. With increasing quality of life and further increases in life expectancy anticipated with the development of curative therapies [36], it is the medical community’s responsibility to ensure meeting the evolving needs and priorities of adults with SCD. While the American Society of Hematology has updated transition summaries to prompt consideration of partner genetic testing [37], most patient education regarding ART is well above the national average reading level [38], limiting the ability of clinicians to fully discuss all options with patients with SCD. Further research is necessary to optimize the health of women with SCD for pregnancy, inform conversations with adults with SCD regarding the risks and benefits of ART, as well as understand the fertility and fetal risks of SCD therapies. Research regarding patient-identified needs in family building planning guidelines is ongoing [39], and patient-centered recommendations could serve as a critical bridge between developing evidence and personalized counseling, by guiding providers on how to lead individualized conversations with patients about their family building risks and options.

Notably, although few participants were initially familiar with the use of ART for people with SCD, several noted that they would be interested in pursuing if it was accessible. However, several participants expressed concerns regarding unknown risks specific to SCD and fertility preservation; as data emerges regarding the risk of SCD complications associated with the process of fertility preservation among women with SCD [21], providers must inform patients of both positive and negative implications of ART. All participants were aware of cost as a barrier, which many cited as a reason they had not seriously considered it. Insurance coverage of IVF and PGT remains a critical barrier to reproductive technology access that will need to be mitigated to allow for wider utilization. Economic analysis shows that IVF with PGT would be cost effective compared to lifetime standard of care treatment for SCD with an incremental savings of over $130,000 [40]. However, use of reproductive technology is low in SCD compared to the general population [41], potentially because of knowledge gaps about this technology for SCD, racial disparities in ART access [42], and the high proportion of adults with SCD insured by Medicaid [41]. Previous work has shown that awareness of reproductive technology can be increased through educational materials [18,35], but other barriers to ART access, including insurance coverage, cost, and fertility subspecialty access, persist. Advocacy for insurance coverage of ART is essential to promoting the ability of adults with SCD to make individualized decisions regarding their family building.

Limitations of our study include that all participants were patients at the same center, so their knowledge and experiences may not be applicable to other centers. There was low baseline knowledge among our participants regarding ART, specifically PGT. While participants were provided some information, this may not accurately reflect an informed decision or their perspectives may change after further reflection. There may have been selection bias with the loss to follow up, given that 10 patients were unable to be contacted to schedule or complete interview. While we had a small sample size which may have limited our ability to identify all themes, we did reach saturation for our identified themes; data saturation was reviewed during analysis regarding participants’ perceptions of family building planning conversations with providers and was met prior to the final interview. However, it is possible that we did not adequately capture all relevant minor themes. Most notably, only 2 male participants completed the interview despite 6 men consenting to participate and 11 men being approached. As participants were lost to follow up and did not formally withdraw, it is not known if there are any gender-specific reasons for the differential completion rates by gender. While it is likely we did not completely assess the gender-specific experiences or perceptions of men with SCD, the male participants did fit within the four identified themes relevant to both sexes and expressed similar perceptions regarding their experiences of family building planning, leading us to believe that several components of family building planning are not sex-specific but rather relevant to both men and women. Further work is necessary to further differentiate experiences of men and women with SCD regarding family building planning, as well as to elicit themes regarding male-specific experiences.

## Conclusion

This study illustrates critical gaps in family building knowledge and counseling for adults with SCD. Participants desired earlier and frequent discussions with their providers about what family building options exist for them and potential outcomes. Further work is needed to fill the gaps identified by patients regarding education about the experience of pregnancy with SCD and resources for partners regarding the importance of SCT testing. Continued efforts to remove barriers to reproductive technology, including access to specialists and insurance coverage, is also necessary to enable access to patients interested in ART. As the life expectancy and quality of life for people with SCD increases, so does the need for critical consideration of their evolving needs, including information for family building.

## Supporting information

S1 File COREG checklist. (PDF)

S2 File Interview guide. (DOCX)
