# Supplementary material for: Experiences of family building counseling and perceptions of reproductive technology among adults with sickle cell disease
Source: PLoS One. 2026 Aug 24;21(8):e0355020. doi: 10.1371/journal.pone.0355020 (PMC13502687; doi:10.1371/journal.pone.0355020)
Supplement: Supplementary file 2 [file pone.0355020.s002.docx]

*My name is ___________. I am a __________ [position] with the Colleges of Medicine and Public Health at The Ohio State University. Our research team is interested in learning about the experiences and opinions of women and men with sickle cell disease about partner testing for sickle cell trait and reproductive planning. The interview should take between 50 to 60 minutes. Please remember that there are no “right” or “wrong” answers. We are interested in your perceptions, ideas, and thoughts. If you are uncomfortable at any time or want to skip a question, let me know. We can stop the interview at any time.*

**CURRENT REPRODUCTIVE STATUS**

What is your age?

How would you describe your gender?

How many biological children do you have?

Would you like to ever(FEMALES – become, MALES – get someone) pregnant to have a child?

Can you describe your current relationship status?

- If in a relationship, do you know your partner’s sickle cell disease or trait status?

**GENERAL FERTILITY INFO**

How do you think your sickle cell disease would affect your ability to have biological children?

- How do you think your treatment for SCD (for example, hydroxyurea or bone marrow transplant) would affect your ability to have biological children?

Can you tell me about what your sickle cell care team has discussed with you about pregnancy or fertility in the past?

- Can you tell me about a previous conversation your healthcare providers (like doctors) have had about future pregnancies or fertility planning with you?

Would you like to talk about reproductive health issues with your healthcare providers more?

- If so, who would you like to discuss this with? (Probes: sickle cell team, primary care, fertility experts, social work, genetic counselors, mental health)
- What topics would you like them to discuss with you?

**PARTNER TESTING**

*As you may know, if both parents have either sickle cell trait or sickle cell disease, their child may develop sickle cell disease. Most people with sickle cell trait don’t have symptoms and may not know that their future children are at risk for sickle cell disease.*

Have you ever asked a partner to be tested for hemoglobin issues, like sickle cell trait, in the past?

- Can you describe an example of one of these conversations?
- How did you feel about that conversation?

Would you be comfortable asking either your [current or future] partner about their sickle cell trait status in the future?

- Tell me more about why or why not.
- What are some reasons you would ask their sickle cell trait status?
- What are some reasons you would not ask their sickle cell trait status?

If they did not know, would you be comfortable asking your [current or future] partner to get tested for sickle cell trait?

- Tell me more about why or why not.
- What are some reasons you would ask them to be tested for sickle cell trait?
- What are some reasons you would not ask them to be tested for sickle cell trait?

Tell me about how your wish to have a biological child with your partner would be affected if you found out they had sickle cell trait?

If your partner wanted to get sickle cell trait testing, what resources do you know of about how to get tested?

**IVF&PGT**

*Next, we are going to talk about testing genes before a baby is born. As you may know, an embryo is made from a woman’s egg and a man’s sperm. This can occur naturally or through something called in vitro fertilization, or IVF. IVF is a process where an embryo is made from a woman’s egg and man’s sperm in a lab and then transferred back into a women’s body in the hopes that the woman gets pregnant. Some people choose to do this if they are having trouble getting pregnant naturally or if they want to try to prevent their child from having a genetic disease. If someone finds out their child is at risk for a genetic disease like sickle cell disease, they could consider preimplantation genetic testing, or PGT. This is a procedure that identifies genetic disorders in embryos created during IVF. After genetic testing, a person can make sure that the baby they become pregnant with does not have the genetic disease they tested for. For example, PGT can be done to determine if a baby will have sickle cell disease or not.*

What, if anything, have you heard about IVF before?

- Who have you discussed IVF with before (ex: healthcare provider, partner, family)

How likely is it you would choose to use IVF if (FEMALES: you MALE: your partner) had trouble getting pregnant?

- Tell me more about why or what not.
- What are some reasons you would be interested?
- What are some reasons you would not be interested?

What, if anything, have you heard about PGT before?

- Who have you discussed PGT with before (ex: healthcare provider, partner, family)

How likely is it you would choose to have genetic testing, like PGT, on an embryo before becoming pregnant if you found out your partner had sickle cell trait?

- Tell me more about why or what not.
- What are some reasons you would be interested?
- What are some reasons you would not be interested?

Fertility procedures like IVF and PGT are not always covered by insurance. Without insurance, IVF with PGT can cost $15,000-$20,000. How would cost play a role in deciding whether to pursue PGT?

- How much money would you be able to spend on a test like this?

What resources do you think would be helpful to learn more about your reproductive options for people with sickle cell disease?

In your opinion, how can your healthcare providers better support people with sickle cell disease with reproductive health concerns?

Are there any other questions I didn’t ask that you think are important?

Are you interested in more information about anything we talked about today?

IF YES, GIVE POST-INTERVIEW HANDOUT
