# Supplementary material for: Experiences of family building counseling and perceptions of reproductive technology among adults with sickle cell disease
Source: PLoS One. 2026 Aug 24;21(8):e0355020. doi: 10.1371/journal.pone.0355020 (PMC13502687; doi:10.1371/journal.pone.0355020)
Supplement: Supplementary file 3 [file pone.0355020.s004.docx]

March 9, 2026

Division of General Internal Medicine

2050 Kenny Rd.

Suite 2335

Columbus, OH 43221

Emily Chenette, PhD

Editor-in-Chief

George Vousden, PhD

Deputy Editor

*PLOS One*

Dear Dr. Chenette and Dr. Vousden,

We are submitting our revised manuscript proposal entitled “Experiences of family building counseling and perceptions of reproductive technology among adults with sickle cell disease.” We are very thankful for the helpful and insightful suggestions from the expert reviewers on our research which has helped us strengthen our study’s findings and importance. We have revised our manuscript in accordance with their comments. Specifically, we are submitting a “point by point” reply to all of them below. We have made significant changes in our description of the methodology and clarified the limitations as well as validity of our work further per the reviewers’ recommendations. We have submitted a copy of the manuscript with tracked changes as requested.

Funding statement: Data collection via REDCap was supported by the National Center for Advancing Translational Sciences, Grant UM1TR004548. The research reported in this publication was supported in part by The Ohio State University (OSU) Center for Health Outcomes in Medicine Scholarship and Service (HOMES). The content is solely the responsibility of the authors and does not necessarily represent the official views of OSU or HOMES. There was no additional external funding received for this study.

Overall, we are grateful to you and the expert reviewers for giving us the opportunity to submit the revised manuscript. We sincerely hope that our study will be of broader interest to the general readership of *PLOS One* and our research can shed light on the family building experiences of people with sickle cell disease. Thank you for your time and consideration. Please reach out with any questions or concerns regarding this manuscript or revisions.

With warm regards,

Rachel D’Amico Gordon, MD, MPH

895 Yard Street

Columbus, OH 43212

Rachel.Gordon@osumc.edu

(513) 277-1646

**Detailed Response to the Editor’s Comments**

*This is prospective research about Experiences of family building counseling and perceptions of reproductive technology among adults with sickle cell disease. Methodology section need to be improved and expand inclusion and inclusion criteria, also to discuss how you chose the adequate sample size while 11 cases is very low.*

**Response: Thank you for this comment; we have expanded the methodology section as well as added a supplemental file to ensure COREQ checklist compliance. We have clarified the determination of eligible participants within the manuscript text. We have also included discussion of data saturation to explain reasoning for 11 participants, as well as expanded our limitations to discuss the impact sample size may have on our study’s external validity.**

*Please include also ethic consideration and consent for both video interview and publication.*

**Response: Thank you for this comment; we have clarified in the Methods section that participants provided consent for video recording of interviews as well as the relevant IRB ID.**

*Please mention clearly why female gender were more than male gender in the study.*

**Response: Thank you for this comment; we have added the gender rates of participants who were consented versus those who completed the interview in the results, as well as the following discussion of gender in our Discussion; “only 2 male participants completed the interview despite 6 men consenting to participate and 11 men being approached. As participants were lost to follow up and did not formally withdraw, it is not known if there are any gender-specific reasons for the differential completion rates by gender. While it is likely we did not completely assess the gender-specific experiences or perceptions of men with SCD, the male participants did fit within the four identified themes relevant to both sexes and expressed similar perceptions regarding their experiences of family building planning, leading us to believe that several components of family building planning are not sex-specific but rather relevant to both men and women. Further work is necessary to further differentiate experiences of men and women with SCD regarding family building planning, as well as to elicit themes regarding male-specific experiences.”**

**Detailed Response to the Reviewers’ Comments**

Reviewer #1 Comments
*1 - Additional details about the Center are needed: is it a National Alliance of Sickle Cell Centers affiliate, how many patients, how many doctors / NPs or PAs? Does the clinic offer referral to MFM, have an REI on campus, access to complex family planning specialists?*

**Response: Thank you for this comment, we have further clarified the details of our medical center with the following addition: “A National Alliance of Sickle Cell Centers affiliate, the included center treats an estimated 400 adults with SCD and has obstetric providers, including specialists in Reproductive Endocrinology and Infertility (REI), within the medical center. “**

*2 - Provide more rigorous methods section per qualitative research reporting standards, i.e. COREQ Guideline. What is training background in qualitative data of research team?*

**Response: Thank you for this comment. We have worked to include further detail in our methods section; to ensure adequate representation of all COREQ Guideline standards, we have included the COREQ checklist as a supplementary file with included page numbers. Specifically related to training, we added the following language into the manuscript: “RDG completed didactic coursework in qualitative analysis and had completed qualitative studies regarding postpartum primary care; KD conducted qualitative analysis for her Master of Public Health [MPH] thesis and was under supervision of RDG.”**

*3 - Participants did not like identify the social ecological framework, therefore any comment they make can be analyzed to fit into the framework, but the abstract needs to be revised: The participants did not identify factors across the social ecological framework, their comments were fit to this analytic frame.*

**Response: Thank you for this comment; we have changed the final line of the Results in Abstract to, “Participants identified factors that impacted their family building plans, which fit across the social ecological model.” We have also clarified similar language in the manuscript Results to ensure it is clear that participant identified features were retroactively fitted to the social ecological model.**

*4 - The rationale for 82% female sample needs explanation; why include 2 men? This is a problem for representativeness and rigor. The limitations needs significant strengthening in this regard unless you can prove that 2 men constitute saturation of themes for this topic which seems implausible given the complexities therein. Overall, the severe limitations of the sample need to be more deeply explored.*

**Response: Thank you for this comment; we have added the following text to the Discussion regarding gender representation of our study. “Only 2 male participants completed the interview despite 6 men consenting to participate and 11 men being approached. As participants were lost to follow up and did not formally withdraw, it is not known if there are any gender-specific reasons for the differential completion rates by gender. While it is likely we did not completely assess the gender-specific experiences or perceptions of men with SCD, the male participants did fit within the four identified themes relevant to both sexes and expressed similar perceptions regarding their experiences of family building planning, leading us to believe that several components of family building planning are not sex-specific but rather relevant to both men and women. Further work is necessary to further differentiate experiences of men and women with SCD regarding family building planning, as well as to elicit themes regarding male-specific experiences.”**

*5 - Discussion may add: There are efforts to include family planning conversations in the adult SCD clinic and recommendations for ASH transition summaries - perhaps too late for 30 year olds with SCD, but not for the aging-into-adult care generation who are positioned to benefit from recent interest in this topic in general. See for example: PMID: 37601884.*

**Response: Thank you for this comment, we have added the following text to the third paragraph of the Discussion, which includes the recommended reference; “While the American Society of Hematology has updated transition summaries to prompt consideration of partner genetic testing,(36) most patient education regarding ART is well above the national average reading level,(37) limiting the ability of clinicians to fully discuss all options with patients with SCD…. Research regarding patient-identified needs in family building planning guidelines is ongoing(38)”**

Reviewer #2

*This manuscript describes an excellent qualitative study of how people with SCD understand the potential fertility implications of SCD and their perceptions of partner testing and ART. This is an important clinical topic with little prior literature available to inform clinicians or patients about this issue. The investigators used a convenience sample of young adults patients with SCD who expressed interest in having a biological child in the future. Semi-structured interviews were conducted with appropriate methodology and analysis strategies. Results are appropriate described in text and Figures. The introduction is concise and appropriately referenced. The discussion appropriately describes the study results in the context of available literature and provides clear recommendations for implementation of study results into clinical practice. Study limitations are acknowledged.*

**Response: Thank you for this comment, we appreciate your acknowledgment of the importance of this novel study.**

*I had two requests for the authors:
1. Please provide the study interview guide as supplementary material.*

**Response: Thank you for this comment; we have provided the interview guide as a supplementary file (S2 File).**

*2. Page 22, first paragraph: Reference #37 does not appear to support the statement, “However, use of reproductive technology is low in SCD compared to other monogenetic conditions,”-please provide a more appropriate reference.*

**Response: Thank you for this comment; we have appended this statement to state, “use of reproductive technology is low in SCD compared to the general population,” which is directly supported by this reference (22.5% of participants reported seeking infertility evaluation compared to about half of women between 15-44 years in the US). While we did find references showing that other monogenetic conditions have higher rates of ART (PMID 40457440), we did not feel this was a reasonable direct comparison as most of the data was from patients with cystic fibrosis, which has specific impacts on fertility such as absence of vas deferens.**
