# Supplementary material for: Experiences of family building counseling and perceptions of reproductive technology among adults with sickle cell disease
Source: PLoS One. 2026 Aug 24;21(8):e0355020. doi: 10.1371/journal.pone.0355020 (PMC13502687; doi:10.1371/journal.pone.0355020)
Supplement: Peer review history

# Peer review: Decision Letter 0

**DOI:** 10.1371/journal.pone.0355020.r001

**Authors:** Rehab Al-Ansari

11 Feb 2026

Dear Dr. Rachel Gordon,

Thank you for submitting your manuscript to PLOS ONE. After careful consideration, we feel that it has merit but does not fully meet PLOS ONE’s publication criteria as it currently stands. Therefore, we invite you to submit a revised version of the manuscript that addresses the points raised during the review process.

Please submit your revised manuscript by Mar 28 2026 11:59PM. If you will need more time than this to complete your revisions, please reply to this message or contact the journal office at plosone@plos.org. When you’re ready to submit your revision, log on to https://www.editorialmanager.com/pone/ and select the 'Submissions Needing Revision' folder to locate your manuscript file.

- A letter that responds to each point raised by the academic editor and reviewer(s). You should upload this letter as a separate file labeled 'Response to Reviewers'.
- A marked-up copy of your manuscript that highlights changes made to the original version. You should upload this as a separate file labeled 'Revised Manuscript with Track Changes'.
- An unmarked version of your revised paper without tracked changes. You should upload this as a separate file labeled 'Manuscript'.

If applicable, we recommend that you deposit your laboratory protocols in protocols.io to enhance the reproducibility of your results. Protocols.io assigns your protocol its own identifier (DOI) so that it can be cited independently in the future. For instructions see: https://journals.plos.org/plosone/s/submission-guidelines#loc-laboratory-protocols. Additionally, PLOS ONE offers an option for publishing peer-reviewed Lab Protocol articles, which describe protocols hosted on protocols.io. Read more information on sharing protocols at https://plos.org/protocols?utm_medium=editorial-email&utm_source=authorletters&utm_campaign=protocols.

We look forward to receiving your revised manuscript.

Kind regards,

Rehab Al-Ansari

Academic Editor

PLOS One

Journal Requirements:

When submitting your revision, we need you to address these additional requirements.

1. Please ensure that your manuscript meets PLOS ONE’s style requirements, including those for file naming. The PLOS ONE style templates can be found at

https://journals.plos.org/plosone/s/file?id=wjVg/PLOSOne_formatting_sample_main_body.pdf and

https://journals.plos.org/plosone/s/file?id=ba62/PLOSOne_formatting_sample_title_authors_affiliations.pdf.

2. Thank you for stating in your Funding Statement:

“Data collection via REDCap was supported by the National Center for Advancing Translational Sciences, Grant UM1TR004548. The research reported in this publication was supported in part by The Ohio State University (OSU) Center for Health Outcomes in Medicine Scholarship and Service (HOMES). The content is solely the responsibility of the authors and does not necessarily represent the official views of OSU or HOMES.”

Please provide an amended statement that declares *all* the funding or sources of support (whether external or internal to your organization) received during this study, as detailed online in our guide for authors at http://journals.plos.org/plosone/s/submit-now.  Please also include the statement “There was no additional external funding received for this study.” in your updated Funding Statement.

Please include your amended Funding Statement within your cover letter. We will change the online submission form on your behalf.

3. We note that you have indicated that there are restrictions to data sharing for this study. For studies involving human research participant data or other sensitive data, we encourage authors to share de-identified or anonymized data. However, when data cannot be publicly shared for ethical reasons, we allow authors to make their data sets available upon request. For information on unacceptable data access restrictions, please see http://journals.plos.org/plosone/s/data-availability#loc-unacceptable-data-access-restrictions.

Before we proceed with your manuscript, please address the following prompts:

a) If there are ethical or legal restrictions on sharing a de-identified data set, please explain them in detail (e.g., data contain potentially identifying or sensitive patient information, data are owned by a third-party organization, etc.) and who has imposed them (e.g., a Research Ethics Committee or Institutional Review Board, etc.). Please also provide contact information for a data access committee, ethics committee, or other institutional body to which data requests may be sent.

b) If there are no restrictions, please upload the minimal anonymized data set necessary to replicate your study findings to a stable, public repository and provide us with the relevant URLs, DOIs, or accession numbers. Please see http://www.bmj.com/content/340/bmj.c181.long for guidelines on how to de-identify and prepare clinical data for publication. For a list of recommended repositories, please see https://journals.plos.org/plosone/s/recommended-repositories. You also have the option of uploading the data as Supporting Information files, but we would recommend depositing data directly to a data repository if possible.

Please update your Data Availability statement in the submission form accordingly.

4. Please be informed that funding information should not appear in the Acknowledgments section or other areas of your manuscript. We will only publish funding information present in the Funding Statement section of the online submission form. Please remove any funding-related text from the manuscript.

5. If the reviewer comments include a recommendation to cite specific previously published works, please review and evaluate these publications to determine whether they are relevant and should be cited. There is no requirement to cite these works unless the editor has indicated otherwise.

6. Please review your reference list to ensure that it is complete and correct. If you have cited papers that have been retracted, please include the rationale for doing so in the manuscript text, or remove these references and replace them with relevant current references. Any changes to the reference list should be mentioned in the rebuttal letter that accompanies your revised manuscript. If you need to cite a retracted article, indicate the article’s retracted status in the References list and also include a citation and full reference for the retraction notice.

Additional Editor Comments (if provided):

Dear Author,

this is prospective research about Experiences of family building counseling and perceptions of reproductive technology among adults with sickle cell disease. methodology section need to be improved and expand inclusion and inclusion criteria, also to discuss how you chose the adequate sample size while 11 cases is very low.

please include also ethic consideration and consent for both video interview and publication.

please mention clearly why female gender were more than female gender in the study.

Best regards

[Note: HTML markup is below. Please do not edit.]

Reviewers' comments:

Reviewer’s Responses to Questions

**Comments to the Author**

1. Is the manuscript technically sound, and do the data support the conclusions?

Reviewer #1: Partly

Reviewer #2: Yes

**********

2. Has the statistical analysis been performed appropriately and rigorously?

Reviewer #1: I Don't Know

Reviewer #2: N/A

**********

3. Have the authors made all data underlying the findings in their manuscript fully available?

The PLOS Data policy

Reviewer #1: No

Reviewer #2: Yes

**********

4. Is the manuscript presented in an intelligible fashion and written in standard English?

Reviewer #1: Yes

Reviewer #2: Yes

**********

Reviewer #1: This is a qualitative study of 11 subjects from a single center in the Midwest.

1 - Additional details about the Center are needed: is it a National Alliance of Sickle Cell Centers affiliate, how many patients, how many doctors / NPs or PAs? Does the clinic offer referral to MFM, have an REI on campus, access to complex family planning specialists?

2 - Provide more rigorous methods section per qualitative research reporting standards, i.e. COREQ Guideline. What is training background in qualitative data of research team?

3 - Participants did not like identify the social ecological framework, therefore any comment they make can be analyzed to fit into the framework, but the abstract needs to be revised: The participants did not identify factors across the social ecological framework, their comments were fit to this analytic frame.

4 - The rationale for 82% female sample needs explanation; why include 2 men? This is a problem for representativeness and rigor. The limitations needs significant strengthening in this regard unless you can prove that 2 men constitute saturation of themes for this topic which seems implausible given the complexities therein. Overall, the severe limitations of the sample need to be more deeply explored.

5 - Discussion may add: There are efforts to include family planning conversations in the adult SCD clinic and recommendations for ASH transition summaries - perhaps too late for 30 year olds with SCD, but not for the aging-into-adult care generation who are positioned to benefit from recent interest in this topic in general. See for example: PMID: 37601884.

Reviewer #2: This manuscript describes an excellent qualitative study of how people with SCD understand the potential fertility implications of SCD and their perceptions of partner testing and ART. This is an important clinical topic with little prior literature available to inform clinicians or patients about this issue. The investigators used a convenience sample of young adults patients with SCD who expressed interest in having a biological child in the future. Semi-structured interviews were conducted with appropriate methodology and analysis strategies. Results are appropriate described in text and Figures. The introduction is concise and appropriately referenced. The discussion appropriately describes the study results in the context of available literature and provides clear recommendations for implementation of study results into clinical practice. Study limitations are acknowledged. I had two requests for the authors:

1. Please provide the study interview guide as supplementary material.

2. Page 22, first paragraph: Reference #37 does not appear to support the statement, “However, use of reproductive technology is low in SCD compared to other monogenetic conditions,”-please provide a more appropriate reference.

**********

what does this mean?). If published, this will include your full peer review and any attached files.

If you choose “no”, your identity will remain anonymous but your review may still be made public.

**Do you want your identity to be public for this peer review?**  For information about this choice, including consent withdrawal, please see our Privacy Policy

Reviewer #1: No

Reviewer #2: **Yes:**  Carlton Dampier MD

**********

[NOTE: If reviewer comments were submitted as an attachment file, they will be attached to this email and accessible via the submission site. Please log into your account, locate the manuscript record, and check for the action link "View Attachments". If this link does not appear, there are no attachment files.]

To ensure your figures meet our technical requirements, please review our figure guidelines: https://journals.plos.org/plosone/s/figures

You may also use PLOS’s free figure tool, NAAS, to help you prepare publication quality figures: https://journals.plos.org/plosone/s/figures#loc-tools-for-figure-preparation.

NAAS will assess whether your figures meet our technical requirements by comparing each figure against our figure specifications.

# Author comment: Author response to Decision Letter 1

**DOI:** 10.1371/journal.pone.0355020.r002

12 Mar 2026

Detailed Response to the Editor’s Comments

This is prospective research about Experiences of family building counseling and perceptions of reproductive technology among adults with sickle cell disease. Methodology section need to be improved and expand inclusion and inclusion criteria, also to discuss how you chose the adequate sample size while 11 cases is very low.

Response: Thank you for this comment; we have expanded the methodology section as well as added a supplemental file to ensure COREQ checklist compliance. We have clarified the determination of eligible participants within the manuscript text. We have also included discussion of data saturation to explain reasoning for 11 participants, as well as expanded our limitations to discuss the impact sample size may have on our study’s external validity.

Please include also ethic consideration and consent for both video interview and publication.

Response: Thank you for this comment; we have clarified in the Methods section that participants provided consent for video recording of interviews as well as the relevant IRB ID.

Please mention clearly why female gender were more than male gender in the study.

Response: Thank you for this comment; we have added the gender rates of participants who were consented versus those who completed the interview in the results, as well as the following discussion of gender in our Discussion; “only 2 male participants completed the interview despite 6 men consenting to participate and 11 men being approached. As participants were lost to follow up and did not formally withdraw, it is not known if there are any gender-specific reasons for the differential completion rates by gender. While it is likely we did not completely assess the gender-specific experiences or perceptions of men with SCD, the male participants did fit within the four identified themes relevant to both sexes and expressed similar perceptions regarding their experiences of family building planning, leading us to believe that several components of family building planning are not sex-specific but rather relevant to both men and women. Further work is necessary to further differentiate experiences of men and women with SCD regarding family building planning, as well as to elicit themes regarding male-specific experiences.”

Detailed Response to the Reviewers’ Comments

Reviewer #1 Comments

1 - Additional details about the Center are needed: is it a National Alliance of Sickle Cell Centers affiliate, how many patients, how many doctors / NPs or PAs? Does the clinic offer referral to MFM, have an REI on campus, access to complex family planning specialists?

Response: Thank you for this comment, we have further clarified the details of our medical center with the following addition: “A National Alliance of Sickle Cell Centers affiliate, the included center treats an estimated 400 adults with SCD and has obstetric providers, including specialists in Reproductive Endocrinology and Infertility (REI), within the medical center. “

2 - Provide more rigorous methods section per qualitative research reporting standards, i.e. COREQ Guideline. What is training background in qualitative data of research team?

Response: Thank you for this comment. We have worked to include further detail in our methods section; to ensure adequate representation of all COREQ Guideline standards, we have included the COREQ checklist as a supplementary file with included page numbers. Specifically related to training, we added the following language into the manuscript: “RDG completed didactic coursework in qualitative analysis and had completed qualitative studies regarding postpartum primary care; KD conducted qualitative analysis for her Master of Public Health [MPH] thesis and was under supervision of RDG.”

3 - Participants did not like identify the social ecological framework, therefore any comment they make can be analyzed to fit into the framework, but the abstract needs to be revised: The participants did not identify factors across the social ecological framework, their comments were fit to this analytic frame.

Response: Thank you for this comment; we have changed the final line of the Results in Abstract to, “Participants identified factors that impacted their family building plans, which fit across the social ecological model.” We have also clarified similar language in the manuscript Results to ensure it is clear that participant identified features were retroactively fitted to the social ecological model.

4 - The rationale for 82% female sample needs explanation; why include 2 men? This is a problem for representativeness and rigor. The limitations needs significant strengthening in this regard unless you can prove that 2 men constitute saturation of themes for this topic which seems implausible given the complexities therein. Overall, the severe limitations of the sample need to be more deeply explored.

Response: Thank you for this comment; we have added the following text to the Discussion regarding gender representation of our study. “Only 2 male participants completed the interview despite 6 men consenting to participate and 11 men being approached. As participants were lost to follow up and did not formally withdraw, it is not known if there are any gender-specific reasons for the differential completion rates by gender. While it is likely we did not completely assess the gender-specific experiences or perceptions of men with SCD, the male participants did fit within the four identified themes relevant to both sexes and expressed similar perceptions regarding their experiences of family building planning, leading us to believe that several components of family building planning are not sex-specific but rather relevant to both men and women. Further work is necessary to further differentiate experiences of men and women with SCD regarding family building planning, as well as to elicit themes regarding male-specific experiences.”

5 - Discussion may add: There are efforts to include family planning conversations in the adult SCD clinic and recommendations for ASH transition summaries - perhaps too late for 30 year olds with SCD, but not for the aging-into-adult care generation who are positioned to benefit from recent interest in this topic in general. See for example: PMID: 37601884.

Response: Thank you for this comment, we have added the following text to the third paragraph of the Discussion, which includes the recommended reference; “While the American Society of Hematology has updated transition summaries to prompt consideration of partner genetic testing,(36) most patient education regarding ART is well above the national average reading level,(37) limiting the ability of clinicians to fully discuss all options with patients with SCD…. Research regarding patient-identified needs in family building planning guidelines is ongoing(38)”

Reviewer #2

This manuscript describes an excellent qualitative study of how people with SCD understand the potential fertility implications of SCD and their perceptions of partner testing and ART. This is an important clinical topic with little prior literature available to inform clinicians or patients about this issue. The investigators used a convenience sample of young adults patients with SCD who expressed interest in having a biological child in the future. Semi-structured interviews were conducted with appropriate methodology and analysis strategies. Results are appropriate described in text and Figures. The introduction is concise and appropriately referenced. The discussion appropriately describes the study results in the context of available literature and provides clear recommendations for implementation of study results into clinical practice. Study limitations are acknowledged.

Response: Thank you for this comment, we appreciate your acknowledgment of the importance of this novel study.

I had two requests for the authors:

1. Please provide the study interview guide as supplementary material.

Response: Thank you for this comment; we have provided the interview guide as a supplementary file (S2 File).

2. Page 22, first paragraph: Reference #37 does not appear to support the statement, “However, use of reproductive technology is low in SCD compared to other monogenetic conditions,”-please provide a more appropriate reference.

Response: Thank you for this comment; we have appended this statement to state, “use of reproductive technology is low in SCD compared to the general population,” which is directly supported by this reference (22.5% of participants reported seeking infertility evaluation compared to about half of women between 15-44 years in the US). While we did find references showing that other monogenetic conditions have higher rates of ART (PMID 40457440), we did not feel this was a reasonable direct comparison as most of the data was from patients with cystic fibrosis, which has specific impacts on fertility such as absence of vas deferens.

Attachment Submitted filename: Editor letter PLOS.docx

# Peer review: Decision Letter 1

**DOI:** 10.1371/journal.pone.0355020.r003

**Authors:** Rehab Al-Ansari

16 Jul 2026

Experiences of family building counseling and perceptions of reproductive technology among adults with sickle cell disease

PONE-D-25-59277R1

Dear Dr. Rachel Gordon,

We’re pleased to inform you that your manuscript has been judged scientifically suitable for publication and will be formally accepted for publication once it meets all outstanding technical requirements.

Within one week, you’ll receive an e-mail detailing the required amendments. When these have been addressed, you’ll receive a formal acceptance letter and your manuscript will be scheduled for publication.

An invoice will be generated when your article is formally accepted. Please note, if your institution has a publishing partnership with PLOS and your article meets the relevant criteria, all or part of your publication costs will be covered. Please make sure your user information is up-to-date by logging into Editorial Manager at Editorial Manager® and clicking the ‘Update My Information' link at the top of the page. For questions related to billing, please contact billing support.

If your institution or institutions have a press office, please notify them about your upcoming paper to help maximize its impact. If they’ll be preparing press materials, please inform our press team as soon as possible -- no later than 48 hours after receiving the formal acceptance. Your manuscript will remain under strict press embargo until 2 pm Eastern Time on the date of publication. For more information, please contact onepress@plos.org.

Kind regards,

Rehab Al-Ansari

Academic Editor

PLOS One

Additional Editor Comments (optional):

Reviewers' comments:

Reviewer’s Responses to Questions

**Comments to the Author**

Reviewer #2: All comments have been addressed

**********

2. Is the manuscript technically sound, and do the data support the conclusions?

Reviewer #2: (No Response)

**********

3. Has the statistical analysis been performed appropriately and rigorously?

Reviewer #2: (No Response)

**********

4. Have the authors made all data underlying the findings in their manuscript fully available?

The PLOS Data policy

Reviewer #2: (No Response)

**********

5. Is the manuscript presented in an intelligible fashion and written in standard English?

Reviewer #2: (No Response)

**********

Reviewer #2: (No Response)

**********

what does this mean?). If published, this will include your full peer review and any attached files.

If you choose “no”, your identity will remain anonymous but your review may still be made public.

**Do you want your identity to be public for this peer review?**  For information about this choice, including consent withdrawal, please see our Privacy Policy

Reviewer #2: No

**********

# Editor report: Acceptance letter

**DOI:** 10.1371/journal.pone.0355020.r004

**Authors:** Rehab Al-Ansari

PONE-D-25-59277R1

PLOS One

Dear Dr. Gordon,

I'm pleased to inform you that your manuscript has been deemed suitable for publication in PLOS One. Congratulations! Your manuscript is now being handed over to our production team.

At this stage, our production department will prepare your paper for publication. This includes ensuring the following:

* All references, tables, and figures are properly cited

* All relevant supporting information is included in the manuscript submission,

* There are no issues that prevent the paper from being properly typeset

You will receive further instructions from the production team, including instructions on how to review your proof when it is ready. Please keep in mind that we are working through a large volume of accepted articles, so please give us a few days to review your paper and let you know the next and final steps.

Lastly, if your institution or institutions have a press office, please let them know about your upcoming paper now to help maximize its impact. If they'll be preparing press materials, please inform our press team within the next 48 hours. Your manuscript will remain under strict press embargo until 2 pm Eastern Time on the date of publication. For more information, please contact onepress@plos.org.

You will receive an invoice from PLOS for your publication fee after your manuscript has reached the completed accept phase. If you receive an email requesting payment before acceptance or for any other service, this may be a phishing scheme. Learn how to identify phishing emails and protect your accounts at https://explore.plos.org/phishing.

If we can help with anything else, please email us at customercare@plos.org.

Thank you for submitting your work to PLOS ONE and supporting open access.

Kind regards,

PLOS ONE Editorial Office Staff

on behalf of

Dr. Rehab Al-Ansari

Academic Editor

PLOS One
